# The Biomechanical Stability of Bone Staples in Cortical Fixation of Tendon Grafts for Medial Collateral Ligament Reconstruction Depends on the Implant Design

**DOI:** 10.1177/03635465221130753

**Published:** 2022-11-02

**Authors:** Adrian Deichsel, Michael J. Raschke, Elmar Herbst, Christian Peez, Simon Oeckenpöhler, Thorben Briese, Jens Wermers, Christoph Kittl, Johannes Glasbrenner

**Affiliations:** * Department of Trauma, Hand and Reconstructive Surgery, University Hospital Münster, Münster, Germany; Investigation performed at the Department of Trauma, Hand and Reconstructive Surgery, University Hospital Münster, Münster, Germany

**Keywords:** medial collateral ligament reconstruction, cortical fixation, bone staples, cyclic loading, load to failure

## Abstract

**Background:**

The promising biomechanical stability of bone staples (BSs) in cortical fixation of tendon grafts for medial collateral ligament (MCL) reconstruction has been revealed by a previous investigation. However, it is currently unknown if the biomechanical stability of cortical fixation of tendon grafts depends on the BS design.

**Purpose:**

To assess the biomechanical stability of cortical fixation of tendon grafts in knee surgery using 4 different BS designs.

**Study Design:**

Controlled laboratory study.

**Methods:**

Cortical fixation of tendon grafts was performed in a porcine knee model at the tibial insertion area of the MCL using 4 different BS designs (n = 40): 8-mm width without spikes (n = 10), 8-mm width with spikes (n = 10), 14-mm width with spikes (n = 10), and 13 mm–wide 4-prong staples with spikes (n = 10). Specimens were mounted in a materials testing machine, and cyclic loading was applied to the tendon graft (500 cycles at 50 and 100 N, respectively), followed by load-to-failure testing. The Kruskal-Wallis test was performed for statistical analysis (*P* < .05), and the post hoc Dunn test was performed for multiple comparisons.

**Results:**

In 4 of 10 specimens with graft fixation using BSs without spikes, slippage of the tendon underneath the BS led to failure of the construct during cyclic loading to 100 N. In the other groups, no fixation failure was observed during cyclic loading. Furthermore, graft fixation using BSs without spikes was found to have significantly more elongation during cyclic loading (8.2 ± 1.9 mm) and a lower ultimate failure load (170 ± 120 N) compared with graft fixation using narrow BSs with spikes (3.4 ± 1.2 mm [*P* < .0001] and 364 ± 85 N [*P* < .05], respectively) and graft fixation using broad BSs with spikes (4.5 ± 1.4 mm [*P* < .05] and 429 ± 67 N [*P* < .001], respectively). No statistical differences in elongation during cyclic loading or ultimate failure load were found between 4-prong staples with spikes (5.0 ± 1.3 mm and 304 ± 85 N) and narrow or broad staples with spikes.

**Conclusion:**

The biomechanical stability of cortical fixation of an MCL graft was comparable between each BS design with spikes (narrow, broad, and 4-prong) in a porcine knee model, whereas BSs without spikes led to failure of the fixation construct during cyclic loading in 4 of 10 specimens and increased elongation and lower ultimate failure loads in the remainder of the group. BSs without spikes may therefore not be recommended for graft fixation.

**Clinical Relevance:**

The use of BSs can help to avoid the conflict of converging tunnels in multiligament reconstruction surgery. An implant design with spikes yields significantly higher biomechanical stability than BSs without spikes.

Different implants are currently used for cortical fixation of tendon grafts in knee surgery, with interference screw fixation being the standard procedure among most of the recently published techniques.^3,5^ However, the creation of the required bony socket may lead to converging tunnels, especially in multiligament knee reconstruction or in combination with osteotomy around the knee.^12,16,17,29^

The use of bone staples (BSs) as an extracortical fixation device offers the advantage of a simplified operative procedure, as BSs do not require a large tunnel for peripheral ligament reconstruction. Other possible advantages of BS fixation are the lower costs per implant in comparison with interference screws or suture anchors as well as better replication of the flat insertion site of native ligaments, such as the tibial medial collateral ligament (MCL) insertion site,^15,27,31^ which to date is mainly reconstructed by semitendinosus, gracilis, or Achilles tendons and interference screw fixation.^5^

A previous investigation revealed the promising biomechanical stability of BSs for cortical fixation of MCL grafts in a porcine knee model without inferiority in comparison with interference screw fixation.^9^ However, currently available BS designs vary regarding the length of their branches, a smooth or spiked surface toward the bone, the width of the implant, and 2- or 4-prong designs.^8,10,18,21,30^ It is currently unknown if the biomechanical stability of cortical fixation of tendon grafts with BSs varies depending on the design of the implant.

Thus, the goal of the present study was to compare the biomechanical stability of cortical fixation of MCL grafts in a porcine knee model using 4 different BS designs. It was hypothesized that (1) the biomechanical stability would be inferior when using BSs with a smooth surface toward the bone in comparison with BSs with spikes and (2) the width of the implant and the number of prongs (2 vs 4 prongs) would not have a significant influence on biomechanical stability.

## Methods

**Figure 1.**
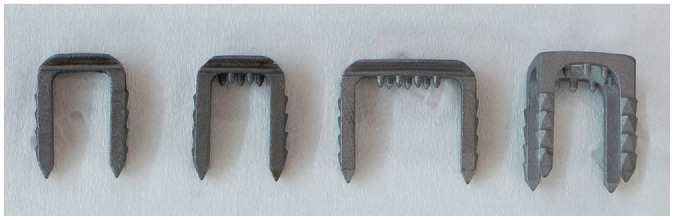
Bone staple designs from left to right: 8-mm width without spikes, 8-mm width with spikes, 14-mm width with spikes, and 4-prong 13 × 13–mm width with spikes.

Fresh porcine tibiae and deep flexor tendons were obtained from a local butcher. The following devices were commercially purchased (Smith & Nephew): Richards Fixation Staple without spikes, 8-mm width, and 15-mm length (narrow and smooth [NSm]); Richards Fixation Staple with spikes, 8-mm width, and 15-mm length (narrow with spikes [NSp]); Richards Fixation Staple with spikes, 14-mm width, and 15-mm length (broad with spikes [BSp]); and Richards Table Fixation Staple with spikes, 13 × 13–mm width, and 16-mm length (4-prong [FpSp]). All devices are shown in Figure 1. No ethical approval was required for this study according to the institutional review board of our institute.

### Test Setup and Graft Fixation

**Figure 2.**
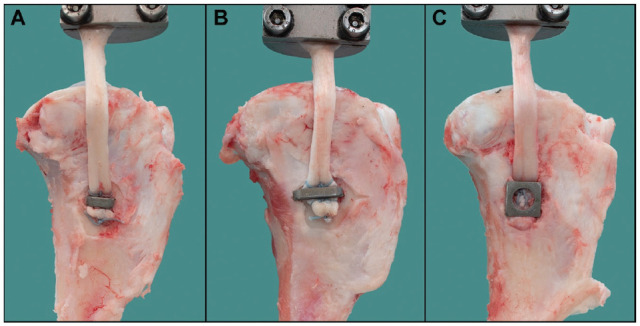
Graft fixation in a porcine knee model using different bone staple designs: (A) 8-mm width with/without spikes, (B) 14-mm width with spikes, and (C) 4-prong 13 × 13–mm width with spikes.

A total of 40 porcine knee specimens were gently defrosted, dissected, and mounted in a cylindrical metal container using synthetic resin (RenCast FC 52/53 A ISO and RenCast FC 53 B Polyol; Gößl & Pfaff). The cylindrical container was firmly attached to the socket of the materials testing machine (Model 8874; Instron). Then, 40 porcine flexor tendons of the knee joint were dissected to a diameter of 6 mm and a length of 80 mm to match the length and thickness of a standard human MCL graft. The diameter of the tendons was measured using a standardized sizing device (±0.5 mm; Karl Storz). The distal end of the tendon graft was sutured using the Krackow technique with 4 stitches on each side^22^ using polyethylene sutures (No. 2 FiberWire; Arthrex). The tendon graft was then fixed under the BS to the center of the tibial insertion area of the MCL, approximately 4 cm distal to the articular surface of the tibia (Figure 2). The BS was inserted perpendicular to the bony surface of the proximal tibia using an orthogonal orientation to the tendon graft.

### Biomechanical Testing

Envelope randomization was used to determine the order of testing. A servohydraulic uniaxial testing machine (Model 8874; Instron) with a 0- to 20-kN sensor was used for cyclic testing. The accuracy of the load cell was ±0.005%, allowing position control with an accuracy of ±0.5% for the testing unit. The cylindrical mount containing the embedded porcine tibia was fixed to the base of the machine with 2 clamps. The proximal end of the graft was fixed to the testing machine using a cryoclamp, leaving 20 mm of free graft between the clamp and the joint line. Before testing, the construct was manually pretensioned with a force of 20 N by positioning the machine’s crossbar. Unidirectional testing in line with the course of the native MCL was performed to simulate a worst-case scenario. The cyclic testing protocol included 500 loading cycles for each step at 50 and 100 N at a rate of 1 Hz based on the loads expected to occur for the native MCL in an anterior cruciate ligament (ACL)–intact (50 N) and ACL-deficient (100 N) knee during normal gait.^23,24,28^ Elongation and load were recorded continuously. Afterward, the construct was loaded to failure at 25 mm/min. Stiffness was determined using the load-elongation curve according to Martin et al.^20^ The mode of failure was macroscopically documented.

### Statistical Analysis

An a priori power analysis showed that a sample size of 10 per group would yield 90% power to detect a difference of 50 N between group means of peak failure load at the *f*≥ 0.8 level based on the standard deviations of MCL graft fixation methods in porcine knee models in previous studies.^9,25^ The normal distribution was evaluated using the Shapiro-Wilk test. The Kruskal-Wallis test was used to compare groups, and the post hoc Dunn test was performed for multiple comparisons. A *P* value <.05 was required to detect significant differences. Statistical analysis was performed using MATLAB (R2020a; MathWorks) and Prism (Version 8; GraphPad Software), and results are presented as mean ± SD.

## Results

Elongation after 500 cycles at 50 N was 5.0 ± 3.2 mm in the NSm group, 1.4 ± 0.5 mm in the NSp group, 2.0 ± 0.5 mm in the BSp group, and 2.3 ± 0.6 mm in the FpSp group. After a further 500 cycles at 100 N, elongation was 8.2 ± 1.9 mm in the NSm group, 3.4 ± 1.2 mm in the NSp group, 4.5 ± 1.4 mm in the BSp group, and 5.0 ± 1.3 mm in the FpSp group. Staples without spikes led to significantly more lengthening in comparison with narrow staples with spikes (*P* < .0001) and broad staples with spikes (*P* < .05).

**Figure 3.**
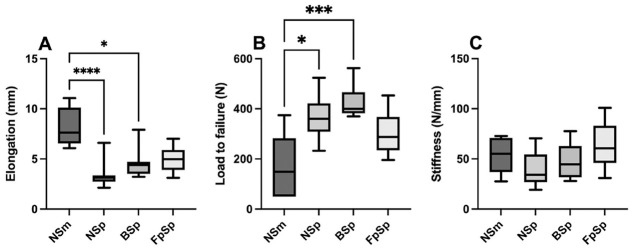
Boxplots presenting the mean (horizontal line), SD (whiskers), and range (box) for (A) elongation after cyclic loading at 100 N, (B) load to failure, and (C) stiffness. Significant difference between groups: *P < .05, ***P < .001, and ****P < .0001. BSp, 14-mm width with spikes; FpSp, 4-prong 13 × 13–mm width with spikes; NSm, 8-mm width without spikes; NSp, 8-mm width with spikes.

The load to failure was significantly higher in the NSp group (364 ± 85 N; *P* < .05) and BSp group (429 ± 67 N; *P* < .001) in comparison with the NSm group (170 ± 120 N). No significant difference was found regarding load to failure of the FpSp group (304 ± 85 N) in comparison with the other groups (*P* < .05). Stiffness was comparable for all BS designs without significant differences between groups. Results are summarized in Figure 3.

The mode of failure was tendon pullout in all specimens of the NSm and FpSp groups, in 9 of 10 specimens of the NSp group, and in 6 of 10 specimens of the BSp group. In 1 specimen of the NSp group and in 4 specimens of the BSp group, the load applied to the tendon led to proximal tilt of the BS, followed by failure of the BS-graft interface. However, elongation and load to failure in these specimens did not differ from the rest of the specimens in the corresponding group. Macroscopically, no slippage at the cryoclamp and no damage to the cortex of the bone were detected in any specimens of all groups after load-to-failure testing.

In 4 of 10 specimens of the NSm group, slippage of the tendon underneath the BS led to failure of the construct during cyclic loading at cycle 439, 449, 430, and 536 of 1000, respectively. Accordingly, elongation at 100 N and peak failure load were reported for only 6 specimens of the NSm group. In the other groups, no construct failure was observed during cyclic loading.

## Discussion

The most important finding of the present study was that the biomechanical stability of cortical fixation of tendon grafts in a porcine knee model was significantly inferior when using BSs without spikes in comparison with narrow or broad BSs with spikes. In contrast, favorable results without significant differences regarding elongation during cyclic loading or ultimate failure load were observed for BSs with spikes when comparing 2 or 4 prongs and 8 mm– or 14 mm–wide 2-prong BSs.

Maximum loading of the MCL during normal gait and while climbing stairs was determined to be 129 N by Morrison.^23,24^ Shelburne et al^28^ simulated loads acting on the native MCL and found them to be around 50 N, with an increase to 114 N in an ACL-deficient knee. In the present study, the mean peak failure loads of graft fixation using BSs with spikes exceeded these loads, whereas BSs without spikes yielded a significantly lower peak failure load (170 ± 120 N), with fixation failure in 4 of 10 specimens during cyclic loading at 50 and 100 N.

On the other hand, failure loads of all tested BS designs were lower than the reported failure loads of the native human MCL, ranging from 465 ± 190 N to 534 ± 85 N.^19,27^ However, with partial load bearing and the use of a knee joint brace aimed to protect the tendon graft, these loads are not expected in the postoperative period after MCL reconstruction.

The use of BSs for graft fixation in MCL reconstruction has been described in a few noncontrolled clinical studies, revealing acceptable or good clinical outcomes.^7,14^ Systematic reviews by DeLong and Waterman^4,5^ examined both MCL repair and reconstruction techniques and were not able to find clinical inferiority of ligament or graft fixation using spiked staples in comparison with other fixation techniques.

In addition to clinical data, there are only a limited number of studies regarding the biomechanical stability of cortical fixation of tendon grafts around the knee joint. Our previous investigation^9^ revealed the promising biomechanical stability of BSs for cortical fixation of MCL grafts in comparison with interference screw fixation and suture anchor fixation in the same porcine knee model: no statistical differences in elongation during cyclic loading or peak failure load were found between narrow BSs with spikes (3.4 ± 1.0 mm and 376 ± 120 N, respectively) and interference screw fixation (3.9 ± 1.2 mm and 313.0 ± 99.5 N, respectively), whereas graft fixation with a single suture anchor was found to have significantly more elongation during cyclic loading (6.4 ± 0.9 mm) and a lower peak failure load (228 ± 49 N).^9^ Omar et al^25^ compared the biomechanical stability of 4.0-mm cancellous screws with different washers as well as single or double titanium suture anchors in a porcine knee model of MCL reconstruction. Spiked polyetheretherketone washers with polyester sutures resulted in the most favorable biomechanical properties for both elongation during cyclic loading (2.9 ± 0.7 mm) and ultimate failure load (470 ± 64 N).^25^ Comparable failure loads were found in the present study for 14 mm–wide staples with spikes (429 ± 67 N). The higher elongation after cyclic loading (eg, 4.5 ± 1.4 mm for 14 mm–wide staples with spikes) might be explained by the intensified cyclic loading protocol of the present study: 500 cycles at 50 N and 500 cycles at 100 N in comparison with 250 cycles at 100 N.^25^

Further available studies are limited to experiments using ACL grafts, which need to resist different loads and are subjected to different angles of force transmission, and the majority of the available studies do not report the exact design of the BS used for graft fixation. Bargar et al^2^ compared 2 BS designs in a canine model with a control of a 6.5-mm cancellous bone screw. At 6 weeks after implantation, the load to failure of the staple constructs was 500 ± 130 N and 540 ± 430 N, respectively. Both groups were inferior to the 6.5-mm cancellous bone screw (1120 ± 490 N).^2^ Letsch^18^ found a maximum load of 508 ± 51 N using a single 2-prong staple and 1210 ± 32 N using 2 staples for cortical fixation of synthetic ACL grafts in human distal femora. BS fixation was compared with cancellous screws with spiked washers for fixation of fascia lata ACL grafts in a goat model by Holden et al.^11^ A single BS without spikes failed at a mean load of 220 ± 16 N, which is comparable with the results of BSs with a smooth surface toward the bone in the present study (170 ± 120 N). Fixation using 2 BSs resulted in an increased failure load of 396 ± 37 N.^11^ Although cancellous bone screws with spiked washers were significantly more stable at time zero, no significant difference was detected at subsequent follow-ups in the goat model, when tendon-to-bone healing had proceeded.^11^

The results of the present study are of clinical relevance, considering that different implant designs of BSs are used in clinical practice for graft fixation,^4,5,8,10^ although the current literature is lacking comparative studies investigating the biomechanical stability of different BS designs. The use of BSs for cortical fixation of tendon grafts can help to avoid confluent tunnels in cases of multiligament reconstruction or when ligament reconstruction is combined with osteotomy.^12,13,29^ Considering the results of the present study, and in concordance with the current literature, the interface between the spiked surface of the BS and suture augmentation of the tendon graft is the key factor for favorable biomechanical stability. This is in accordance with the fact that 4 of 10 specimens using BSs without spikes failed during cyclic loading to 100 N by graft slippage in the present study.

In clinical practice, biomechanical stability may be increased by additional suture fixation of the tendon graft to surrounding soft tissue. Furthermore, graft fixation using a second implant may further improve fixation strength.^11,18^ On the other hand, implant-associated complaints with subsequent hardware removal should be considered when using BSs, and growth plate disturbance by BS implantation must be avoided in adolescent patients.

Several limitations must be considered when transferring the results of the present study to clinical practice. Although previous studies have shown that the porcine knee is suitable for use in biomechanical investigations,^26^ and porcine flexor tendons possess similar biomechanical properties as human semitendinosus tendons,^6^ bone density in the porcine model is significantly higher in comparison with the human knee joint.^1^ Exact descriptive data of the porcine specimens were not obtainable. The biomechanical stability of cortical fixation of tendon grafts is assumed to depend on bone density; therefore, staple pullout or tilt may occur with a higher incidence in human bone with lower density. Second, some of the porcine flexor tendons were trimmed to a diameter of 6 mm, which may have altered their biomechanical properties. Third, unidirectional testing was chosen to simulate a worst-case scenario but may not mimic forces acting in vivo, and the load to failure was applied after cyclic loading of the construct. Fourth, biomechanical testing was a simulation of forces acting at time zero, and biological factors and graft healing were not taken into account. Finally, only BSs of 1 manufacturer were tested; several other implants are available and may present different ultimate strength and stiffness values.

## Conclusion

The biomechanical stability of cortical fixation of an MCL graft was comparable between each BS design with spikes (narrow, broad, and 4-prong) in a porcine knee model, whereas BSs without spikes led to failure of the fixation construct during cyclic loading in 4 of 10 specimens and increased elongation and lower ultimate failure loads in the remainder of the group. BSs without spikes may therefore not be recommended for graft fixation.
